# Supplementary material for: Quiescent OXPHOS-High Triple-Negative Breast Cancer Cells That Persist After Chemotherapy Depend on BCL-XL for Survival
Source: Cells. 2025 Oct 8;14(19):1557. doi: 10.3390/cells14191557 (PMC12524137; doi:10.3390/cells14191557)
Supplement: Supplementary file 1 [file cells-14-01557-s001.zip › cells-3858301-supplementary.pdf]

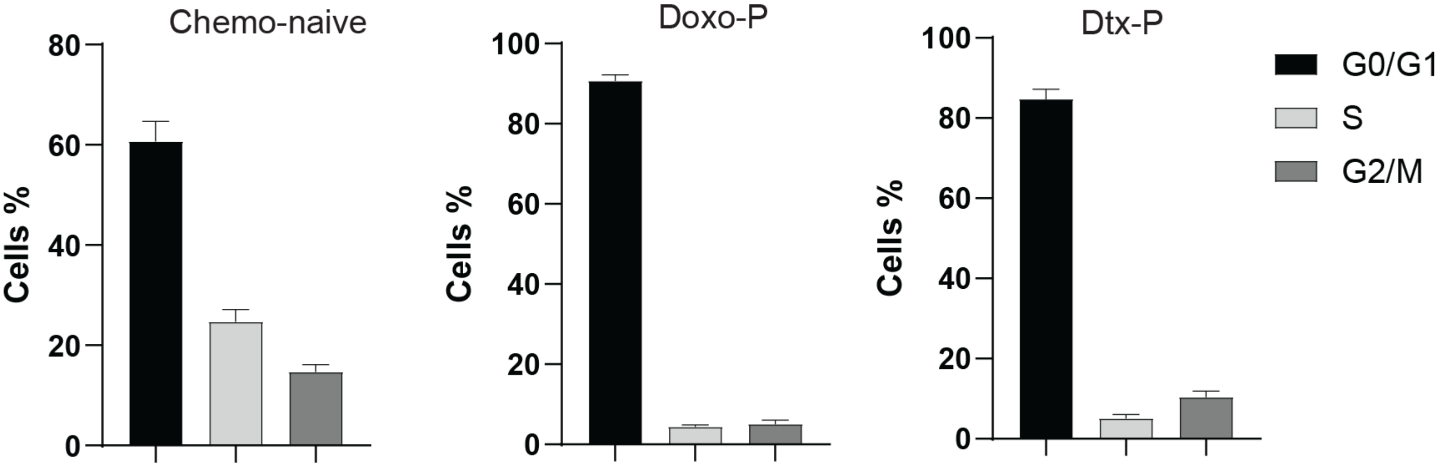

Figure S1. **Quiescent state of chemotherapy-persistent TNBC cells.** Cell cycle analysis of chemo-naïve and chemotherapy-persistent MDAMB-231 cells 3 days after 2h pulse treatment with indicated drugs.

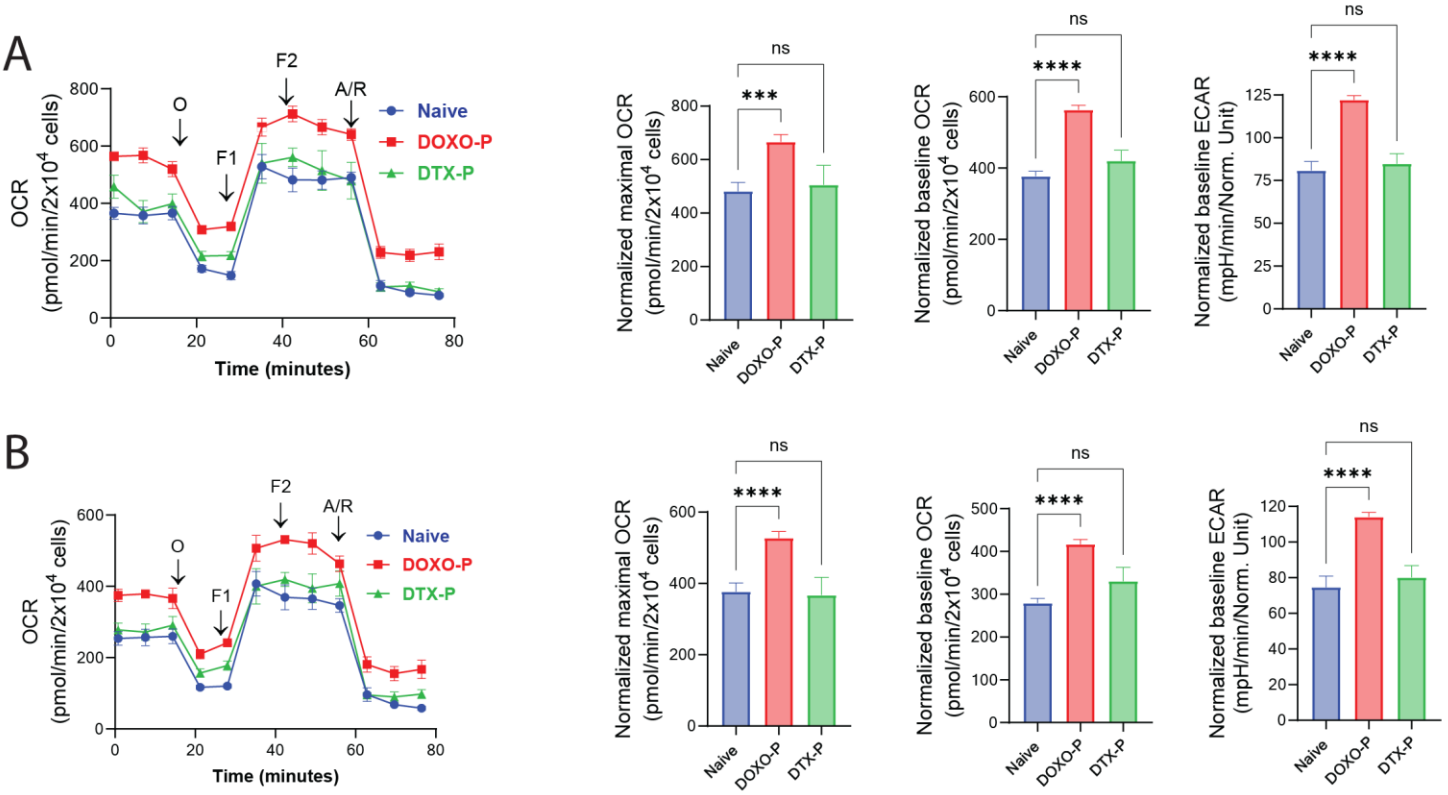

Figure S2. **Upregulated mitochondrial respiration in Doxo-P TNBC cells. A-B)** Mitochondrial respiration in chemo-naïve and chemotherapy-persistent HCC1806 (A) and SUM159 (B) TNBC cells measured using the Seahorse analyzer; values normalized to cell number; ****P < 0.0001, ***<0.001, **<0.01, *<0.05, one-way ANOVA.

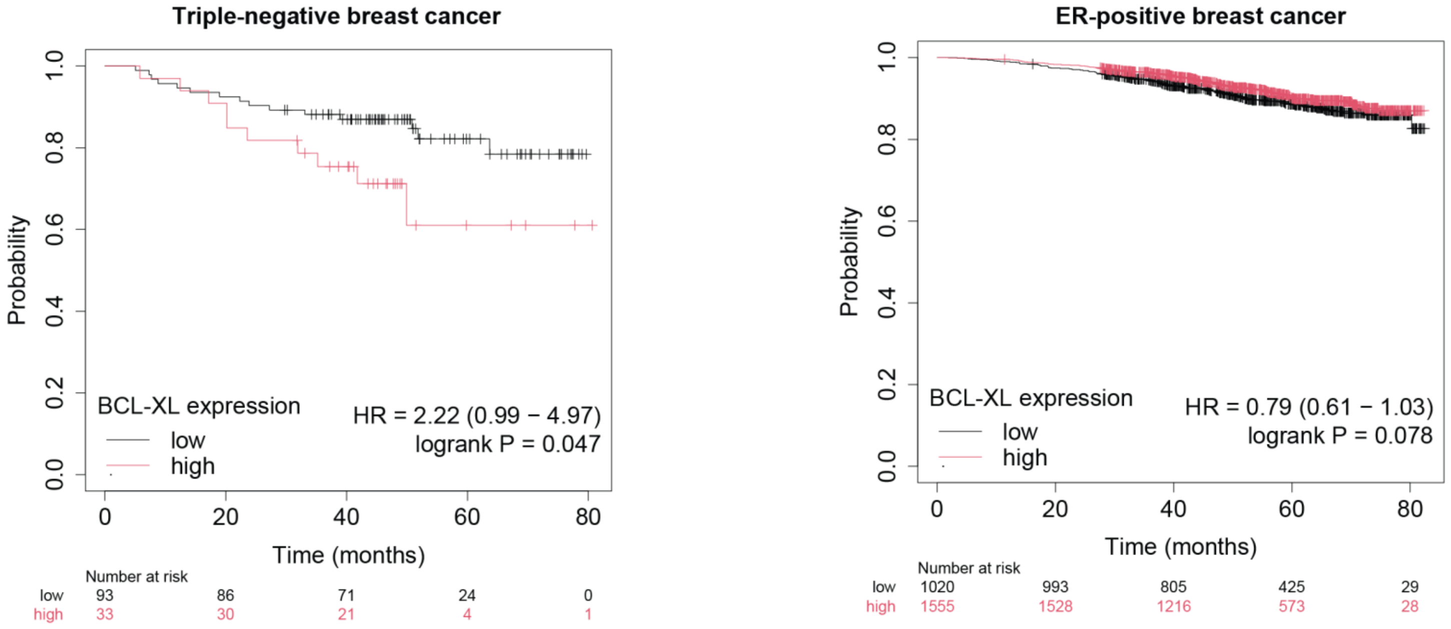

Figure S3. **Prognostic role of BCL-XL in breast cancer.** Correlation of BCL-XL expression with Overall Survival in triple-negative and ER-positive breast cancer patients. KM-plotter online survival analysis tool [41] accessed on July 31st 2025.

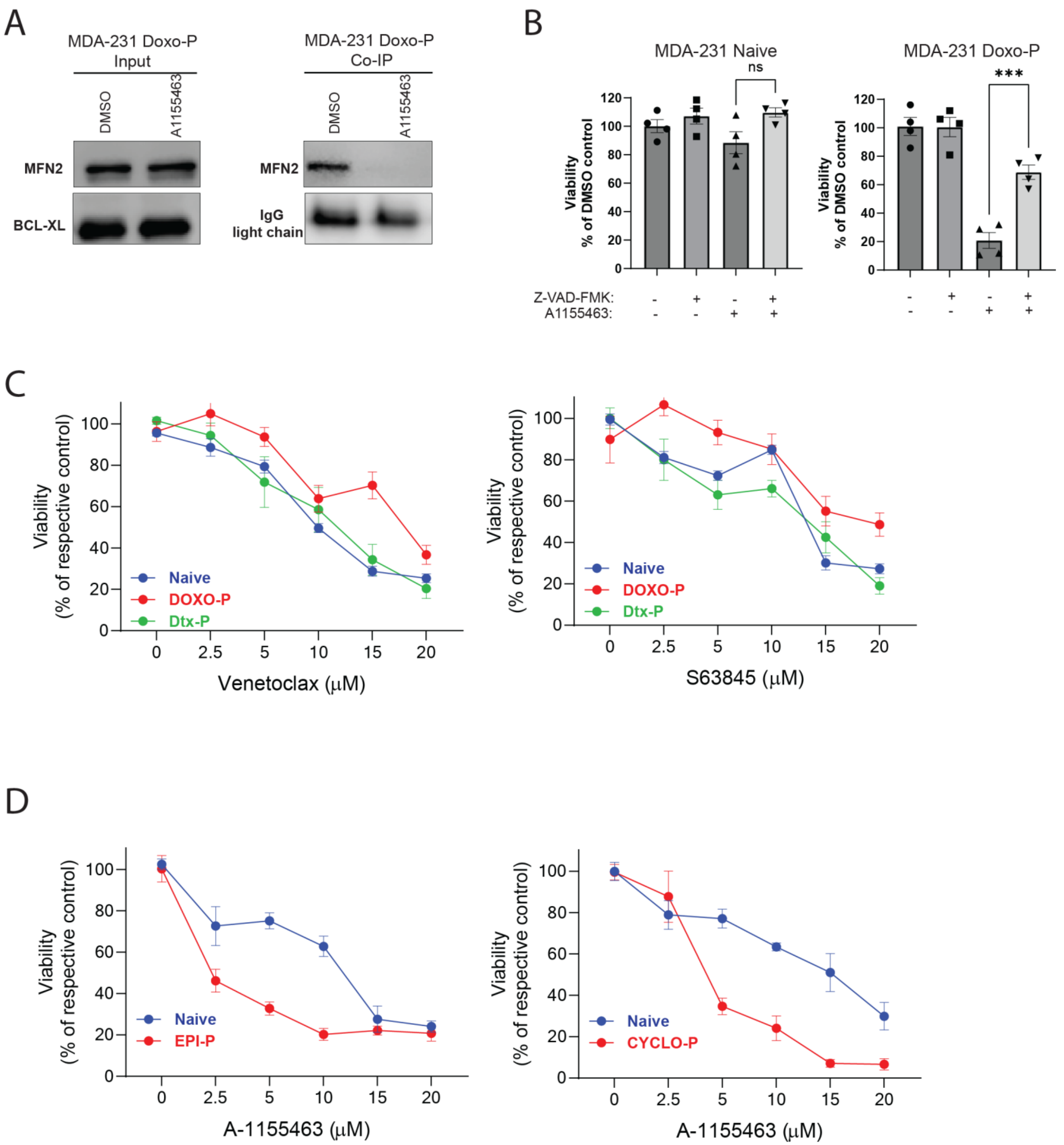

Figure S4. **Phenotypic characterization of chemotherapy-persistent MDAMB-231 cells treated with inhibitors of BCL2, MCL1 and BCL-XL. A)** Immunoprecipitation of BCL-XL in Doxo-P TNBC cells treated with A-1155463 (10μM, 1h exposure) and analysis by western blot using antibodies against MFN2 and BCL-XL (the lower band in the Co-IP blot likely represents overlapping BCL-XL and IgG light chain proteins). **B)** Viability of chemo-naïve and Doxo-P MDAMB-231 cells after co-treatment with BCL-XL inhibitor A-1155463 (5μM) and pan-caspase inhibitor Z-VAD-FMK (20μM) for 24h. **C)** Sensitivity of chemo-naïve, Doxo-P and Dtx-P MDAMB-231 cells to BCL2 inhibitor venetoclax and MCL1 inhibitor S63845. **D)** Sensitivity of chemo-naïve, epirubicin-persistent (Epi-P) and cyclophosphamide-persistent (Cyclo-P) MDAMB-231 cells to BCL-XL inhibitor A1155463.

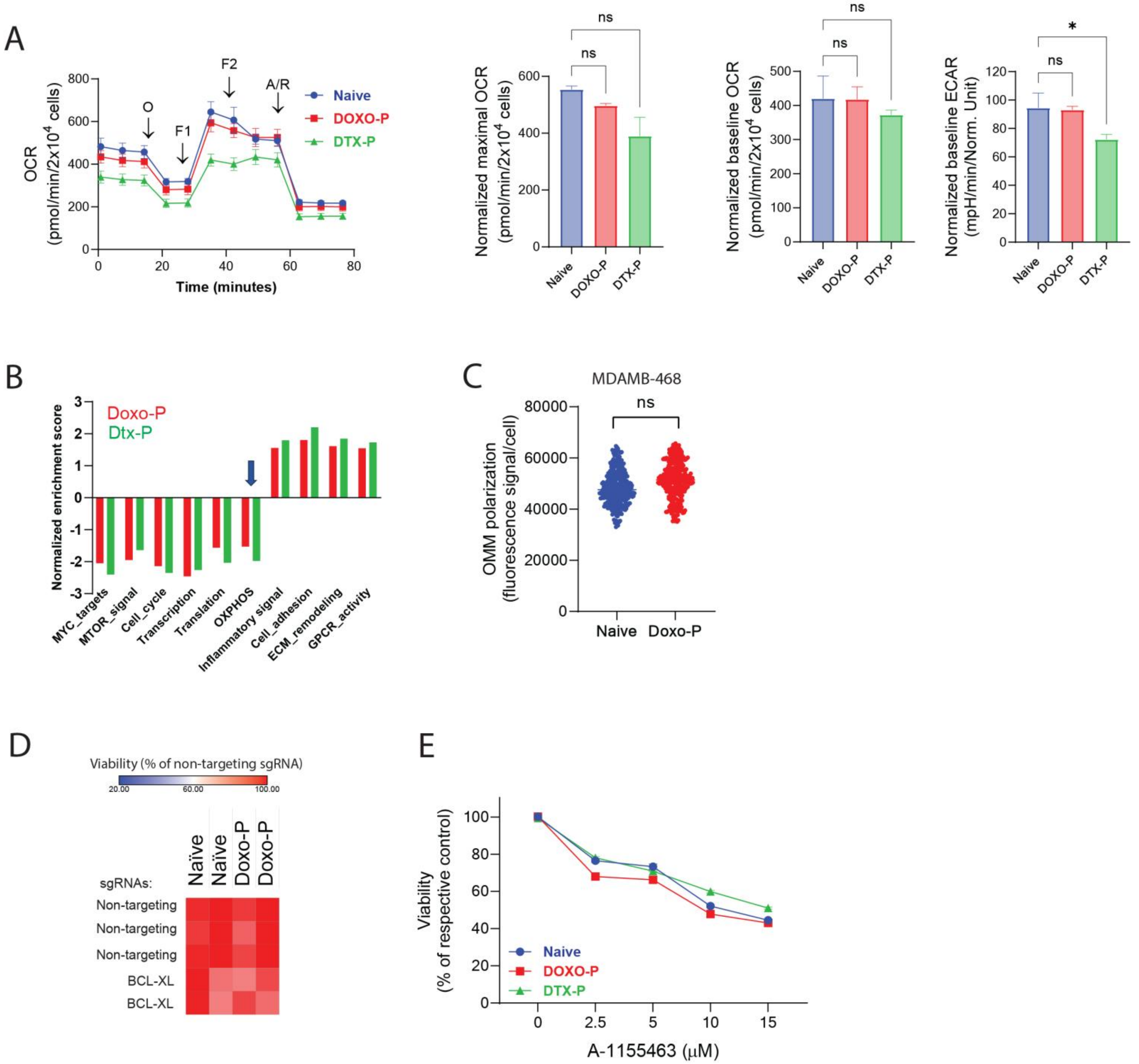

Figure S5. **Doxo-P MDAMB-468 cells have low OXPHOS levels and do not depend on BCL-XL for survival. A)** Mitochondrial respiration in chemo-naïve and chemotherapy-persistent MDAMB-468 cells measured using the Seahorse analyzer; values normalized to cell number. **B)** GSEA analysis of transcriptional changes in MDAMB-468 cells surviving treatment with doxorubicin (Doxo-P) or docetaxel (Dtx-P), compared to chemo-naïve counterparts. **C)** OMM polarization levels in individual cells from chemo-naïve and Doxo-P MDAMB-468 cells, measured using the JC-1 staining assay. **D)** Viability of chemo-naïve and Doxo-P MDAMB-468 after *BCL-XL* gene knockout. **E)** Sensitivity of chemo-naïve, Doxo-P and Dtx-P MDAMB-468 cells to BCL-XL inhibitor A1155463.

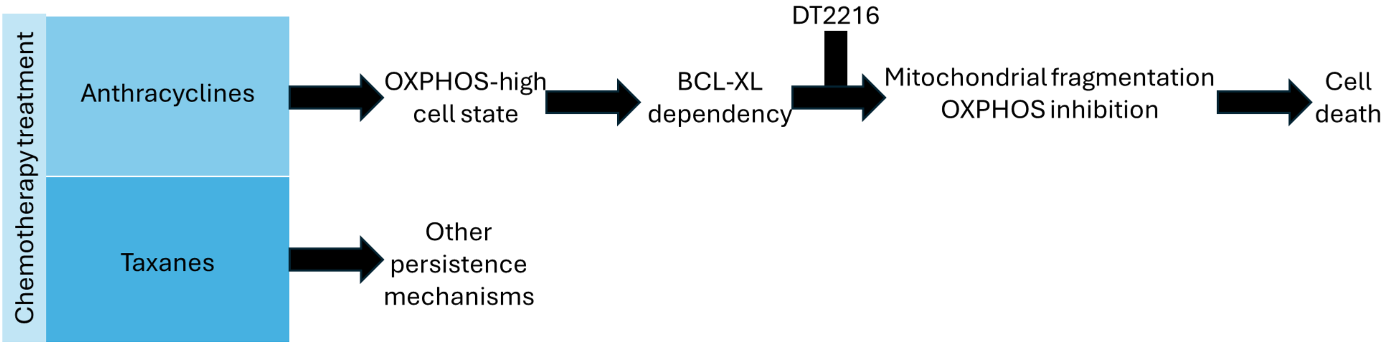

Figure S6. **Schematic representation of chemotherapy persistence model.**
